# MicroRNA profiling reveals age-dependent differential expression of nuclear factor κB and mitogen-activated protein kinase in adipose and bone marrow-derived human mesenchymal stem cells

**DOI:** 10.1186/scrt90

**Published:** 2011-11-14

**Authors:** Amitabh C Pandey, Julie A Semon, Deepak Kaushal, Regina P O'Sullivan, Julie Glowacki, Jeffery M Gimble, Bruce A Bunnell

**Affiliations:** 1 Center for Stem Cell Research and Regenerative Medicine, School of Medicine, Tulane University, New Orleans, LA, USA; 2 Division of Bacteriology and Parasitology, Tulane National Primate Research Center, Covington, LA, USA; 3 Department of Orthopedic Surgery, Brigham and Women's Hospital, Harvard Medical School, Boston, MA, USA; 4 Stem Cell Laboratory, Pennington Biomedical Research Center, Louisiana State University System, Baton Rouge, LA, USA; 5 Department of Pharmacology, School of Medicine, Tulane University, New Orleans, LA, USA; 6 Division of Regenerative Medicine, Tulane National Primate Research Center, Covington, LA, USA

## Abstract

**Introduction:**

Mesenchymal stem cells (MSCs) play a central role in mediating endogenous repair of cell and tissue damage. Biologic aging is a universal process that results in changes at the cellular and molecular levels. In the present study, the role of microRNA (miRNA) in age-induced molecular changes in MSCs derived from adipose tissue (ASCs) and bone marrow (BMSCs) from young and old human donors were investigated by using an unbiased genome-wide approach.

**Methods:**

Human ASCs and BMSCs from young and old donors were cultured, and total RNA was isolated. The miRNA fraction was enriched and used to determine the expression profile of miRNA in young and old donor MSCs. Based on miRNA expression, differences in donor MSCs were further investigated by using differentiation assays, Western blot, immunocytochemistry, and bioinformatics.

**Results:**

Biologic aging demonstrated reduced osteogenic and adipogenic potential in ASCs isolated from older donors, whereas cell size, complexity, and cell-surface markers remained intact with aging. Analysis of miRNA profiles revealed that small subsets of active miRNAs changed secondary to aging. Evaluation of miRNA showed significantly decreased levels of gene expression of inhibitory kappa B kinase (IκB), interleukin-1α, inducible nitric oxide synthase (iNOS), mitogen-activated protein kinase/p38, ERK1/2, c-fos, and c-jun in MSCs from older donors by both bioinformatics and Western blot analysis. Nuclear factor kappa B (NF-κB), *myc*, and interleukin-4 receptor mRNA levels were significantly elevated in aged cells from both the adipose and bone marrow depots. Immunocytochemistry showed nuclear localization in young donors, but a cytosolic predominance of phosphorylated NF-κB in ASCs from older donors. Western blot demonstrated significantly elevated levels of NF-κB subunits, p65 and p50, and AKT.

**Conclusions:**

These findings suggest that differential expression of miRNA is an integral component of biologic aging in MSCs.

## Introduction

Age-related changes occur in all biologic systems, from the phenotypic to the molecular level, leading to activation and deactivation of cellular pathways. Recent studies suggest that mesenchymal stem cells (MSCs) are subject to changes that accompany biologic aging [1-3]. MSCs, also known as mesenchymal stromal cells, are a multipotent, heterogeneous population of cells that possess the ability to differentiate along a variety of cell lineages. MSCs have been isolated from numerous tissue sources, including the bone marrow (BMSCs) and adipose tissue (ASCs), and have been shown to retain the ability to differentiate into numerous terminally differentiated cell types, including bone, cartilage, fat, muscle, and skin [4-6]. Studies also have investigated the role of MSCs as therapeutic agents in many disease states [4,7]. It has been suggested that populations of MSCs are depleted with age and that reduction in MSC pools contributes to human aging and the onset of age-related disease processes [8,9].

Biologic aging can affect not only the absolute numbers of MSCs, but also the expression profile of these cells [9-11]. Indeed, MSCs appear to be as susceptible as other cells to molecular alterations that result from *in vivo *biologic aging [2,3,12]. It has been suggested that MSCs isolated from older donors have an overall decline in differentiation potential or may show a greater propensity toward adipogenesis than toward other cell fates; however, most of these studies focused solely on BMSCs [1,2,13]. Other reports allude to a more complex pattern of events, especially with regard to the adipogenic potential of MSCs and aging [14]. However, the changes exhibited by MSCs due to aging have not been fully delineated. Moreover, the effect of aging on the therapeutic potential of MSCs for regenerative medicine remains to be fully elucidated. It has been suggested that microRNAs (miRNAs) play an integral role in the regulation of aging and subsequent changes associated with the aging process [15-18]. Specifically, miRNAs, which are small 19- to 27-nucleotide (nt) RNA fragments, function in the translational regulation of gene expression. They are members of a large class of small noncoding RNAs. Degradation and repression of target mRNA transcripts are the primary mechanisms whereby miRNAs regulate gene expression and influence cellular processes and signaling mechanisms [19,20]. It has been estimated that approximately two thirds of the whole mammalian genome may be influenced by translational regulation of gene expression by miRNA activity [21]. Indeed, miRNAs appear to be integral regulators of gene expression, influencing processes that include aging, apoptosis, cancer, and inflammation [15,22,23].

Recent studies have investigated the role of miRNAs in MSCs as they progress from undifferentiated states to differentiated end-cell fates, in a variety of species [24,25]. No investigations to date, however, have delved into the effects of biologic age-induced miRNA changes on MSCs. Given the potential of MSCs as cellular therapeutic agents, it is imperative to gain a full understanding of the effects of biologic aging on MSC properties, as well as the potential benefits and risks of using older or younger donor MSCs as treatment modalities.

In the current study, the alterations in the miRNA profiles of MSCs isolated from old and young donors were investigated, and significant downstream alterations that may be manifested secondary to biologic aging have been identified.

## Materials and methods

### Materials

Cell-culture materials, including Hank's buffered saline solution (HBSS), α-modified Eagle's medium (α-MEM), L-glutamine, penicillin/streptomycin, phosphate-buffered saline (PBS), and trypsin/EDTA were obtained from Invitrogen (Carlsbad, CA). Fetal bovine serum (FBS) was purchased from Atlanta Biological (Atlanta, GA). All reagents for miRNA and mRNA arrays were obtained from SABiosciences (Frederick, MD), including whole human genome miRNA array, signal-transduction pathway finder arrays, and RT^2 ^First Strand Kits. Protease and phosphatase inhibitor cocktails, RIPA buffer, and BCA protein quantification kits were ordered from Thermo Scientific (Rockford, IL). Western blot reagents were obtained from Invitrogen; these included loading buffer, reducing agent, 4% to 12% Bis-Tris SDS-PAGE gels, iblot nitrocellulose membrane and blotting components, and chemiluminescence HRP developer kits. The chemiluminescence blocker was acquired from Millipore (Billerica, MA). All antibodies, both primary and secondary, were obtained from Santa Cruz Biotechnology (Santa Cruz, CA). All other chemicals used were molecular biology reagent grade.

### Mesenchymal stem cell isolation and expansion

ASCs and BMSCs were isolated and expanded as previously reported [26]. In brief, ASCs were isolated from subcutaneous white adipose tissue. BMSCs were obtained from the iliac crest or marrow discarded during orthopedic procedures [2,26]. In brief, BMSCs were separated over a Ficoll gradient by centrifugation for 30 minutes at 1,800 *g*. Similarly, ASCs were isolated from subcutaneous white adipose tissue, after which the adipose tissue was digested with 0.075% collagenase for 30 minutes at 37°C to isolate ASCs. Subsequently, all cells were washed in HBSS or PBS, and then centrifuged at 300 *g *(ASC) or 1,000 *g *(BMSC) for 10 minutes. The pelleted nucleated cells were cultured overnight in a humidified atmosphere at 37°C with 5% CO_2 _in complete culture medium (CCM) for BMSCs, which consisted of α-MEM, 20% FBS, 1% L-glutamine, and 1% penicillin/streptomycin or stromal medium (SM) for ASCs (DMEM/F12, 10% FBS, 1% penicillin/streptomycin/amphotericin). The CCM was replaced every third day until the cells reached 70% to 80% confluence. All cells used were between passages 3 and 5 and identified as young donors (younger than 50 years) or old donors (older than 50 years). Young donors of ASCs were an average age of 31.5 ± 10.4 years, whereas BMSCs were 31.5 ± 8.7 years; old donors of ASCs were an average age of 63 ± 6.0 years, and old donors of BMSCs were an average age of 56.3 ± 5.0 years. Investigators were blinded to donor information about the ASCs and BMSCs; however, donor age, race, and other selected demographics were obtained. All donor groups had significant differences in age, but no other significant demographic differences, including BMI. All cells were isolated after review and approval by the institutional review board (IRB) of Tulane University School of Medicine, Pennington Biomedical Research Center, or Brigham and Women's Hospital, with informed patient consent.

### Flow cytometry

Flow cytometry was performed as previously described [27]. After MSCs were 70% confluent, CCM was aspirated, and cells were washed twice with PBS. Cells were harvested with trypsin, and resuspended in PBS for analysis. Cells were assessed for size by using forward and side light-scatter measurements. In addition, all cells were characterized by examination for cell-surface markers by using an extensive panel of antibodies developed by our Center over the past several-years period [28]. Both BMSCs and ASCs expressed known MSC markers including CD29, CD44, CD90, CD105, CD166, and HLA class I. Both cell types were negative for lymphohematopoietic lineage markers, such as CD3, CD34, CD45, CD11b, and CD19.

### Differentiation assay

The ability of MSCs to differentiate along osteogenic and adipogenic lineages was adapted from our previously described methods [29,30]. In brief, cells were plated in 24-well plates and cultured in CCM until they attained 70% confluence. Culture medium was then aspirated and replaced with differentiation-specific medium. Osteogenic differentiation was assessed by staining for bone mineralization with Alizarin Red. Assessment of lipid inclusions, which indicate differentiation to adipocytes, was done by staining with Oil Red O solution, followed by microscopic examination on a Nikon Eclipse TE200 microscope (Nikon Instruments, Melville, NY). Differentiation was quantified as previously described [31]. In brief, after cells were stained, they were destained by using 10% cetylpyridinium chloride for Alzarian Red and isopropyl alcohol for Oil Red O. Collected samples were then analyzed by using a microplate reader at 580 nm to assess the optical density (OD) of the collected samples. Sample OD values were then normalized to protein concentrations of the differentiated cells.

### miRNA profiling arrays of MSCs

The microRNA profiling was performed by using the SABiosciences quantitative polymerase chain reaction (qPCR) array platform according to the protocols of the SABiosciences service core. In total, 16 donors, comprising four young and four old donors of ASCs and BMSCs, respectively, were analyzed with the whole human genome miRNA qPCR array. Samples of ASCs and BMSCs were pelleted and quantified to contain approximately 1 × 10^7 ^cells per donor. Cell pellets were flash frozen with liquid nitrogen and sent to the SABiosciences service core (Frederick, MD) for total RNA isolation, miRNA enrichment, qPCR-based array, and data report. The miRNA profile was analyzed for hierarchic clustering of miRNA by using Genesis to generate heatmaps (Genesis, Austria) [32]. Genesis was also used to cluster donor profiles to identify individual donor variability.

### Target generation from miRNA data

In addition to targets for miRNA action that have been validated in reported studies, potential targets for miRNA action were predicted by using putative targets generated from TargetScan Human V5.1. TargetScan Human predicts putative targets based on factors that include sequence homology, predicted biologic function, and verified targets. The predicted targets were ranked in order of conserved sequences and the prospect of regulation of gene expression via miRNA activity by using a context score, and previous studies have shown that context scores less than -0.3 are commonly considered biologically relevant [33]. The predicted gene targets were analyzed with Ingenuity Pathways Assessment (IPA) and the Ingenuity Knowledgebase (Version 8.7, Ingenuity Systems, Redwood City, CA).

### Bioinformatics analysis of generated miRNA targets

Targets generated by TargetScan were entered into IPA to delineate further the interactions and functions of miRNA on mRNA levels and the protein expression. IPA also generated lists of focus molecules, which were defined by direct and indirect molecules. Direct molecules were those input directly into IPA, such as the targets from TargetScan. Indirect molecules were those that have been described in previous studies, are commonly associated with the direct molecules, and are part of the Ingenuity Knowledgebase. IPA was used to gain insight into the interacting networks of the focus molecules, canonic pathway involvement, biologic functions, and cellular processes influenced by miRNA actions. Canonic pathways are those pathways in which miRNA may play integral roles in regulation. IPA analysis generates these canonic pathways based on the integration of inputted mRNA targets that were generated from significantly changed miRNA. Biologic functions are generated by IPA analysis investigating which physiological and pathological functions may be influenced by the inputted targets. Both the canonic pathways and biologic functions are then evaluated for statistical significance.

### mRNA arrays of MSCs

Signal-transduction qPCR-based array was obtained from SABiosciences and was performed according to the manufacturer's instructions. In brief, total RNA isolated for the miRNA arrays was obtained from two ASC samples based on clustering of the miRNA profile of donors. Representative samples from young and old donors were selected for analysis. Total RNA samples (500 ng) were processed for cDNA synthesis by using a RT^2 ^First Strand Kit according to the manufacturer's protocol. The Signal Transduction Pathway Finder array from SABiosciences was performed on a Bio-Rad CFX96 real-time PCR machine (Bio-Rad, Hercules, CA), according to the manufacturer's instructions. Data from the array were analyzed with the SABiosciences software by observed Ct values and determining the relative fold change as compared with housekeeping genes. Housekeeping genes used as internal controls included β_2_-microglobulin, hypoxanthine phosphoribosyltransferase 1, ribosomal protein L13a, glyceraldehyde-3-phosphate dehydrogenase, and β-actin.

### Subcellular localization of NF-κB subunits by immunocytochemistry

Representative ASCs from old and young donors were selected based on hierarchic clustering of donors from miRNA analysis. Cells were plated at a density of 1 × 10^4 ^cells per 0.4 cm^2^. The next day, cells were fixed for 15 minutes at room temperature, washed, permeabilized for 5 minutes at room temperature, and washed again. Cells were incubated overnight at 4°C with a 1:200 dilution of p50 and p65 subunits of NF-κB, followed by 1 hour at room temperature with a 1:1,000 dilution of a goat anti-rabbit FITC secondary antibody. Slides were counterstained with DAPI, photographed with a Leica DMRXA2 microscope, and rendered with Slidebook software (Denver, CO).

### Cell lysate and protein isolation

ASCs were grown in 15-cm^2 ^dishes to 70% confluence at 37°C, as described earlier. Cells were washed twice with PBS and harvested with trypsin/EDTA. Cells were centrifuged, collected, flash frozen in liquid nitrogen, and stored at -80°C. Cell pellets were lysed in RIPA buffer (reference for RIPA buffer) containing protease and phosphatase inhibitor cocktails. Cell lysate was incubated on ice for 15 minutes with intermittent agitation, and clarified by centrifuging at 14,000 *g *for 15 minutes at 4°C. The supernatant was collected, and the protein concentration of samples was assessed by using a BCA kit and microplate reader (Optica). Protein samples were stored at -80°C until assayed.

### Western blot analysis

Aliquots of 10 μg of total cellular protein from ASCs from old and young donor sample buffered with reducing agent were reduced, boiled, and placed on ice. Bis-Tris 4% to 12% SDS-PAGE gels were used to separate proteins of interest, which were transferred to a nitrocellulose membrane. The membranes were developed with chemiluminescence HRP developer according to the manufacturer's directions, and then immediately imaged by using the Image Quant LAS 4000 imager (GE Healthcare Life Sciences, Piscataway, NJ). Analysis and densitometry were performed with the Image Quant software and Image Quant imager.

### Statistical analysis

The miRNA data were analyzed for relative fold changes from Ct values by using the 2^-ΔΔCt ^method [34]. Statistical significance was determined by comparisons of relative fold regulation of older versus younger donors, with a *P *value < 0.05 indicating significance. Data for miRNA were normalized to the average of four small RNA molecules, SNORD48, SNORD47, SNORD44, and RNU6. For analysis of mRNA data, relative fold changes were determined from Ct values by using the 2^-ΔΔCt ^method. Statistical significance was determined by comparison of fold regulation of older with younger donors, with fold regulation more than equal or less than equal to ± 2, indicating significance. Additionally, *P *values were calculated for the miRNA expression profiles, and a *P *< 0.05 was used to identify those miRNAs whose fold changes were significant. IPA analysis used the right-tailed Fisher Exact test to calculate *P *values, with a *P *< 0.05 indicating significance of association to predicted targets and potential involvement in canonic pathways, biologic function, and networks assessment. All other data were analyzed with Sigma Plot by using a Student test, with *P *< 0.05 indicating statistical significance. All data are presented as mean ± standard error of the mean (SEM).

## Results

### Characterization of MSCs

**Figure 1:**
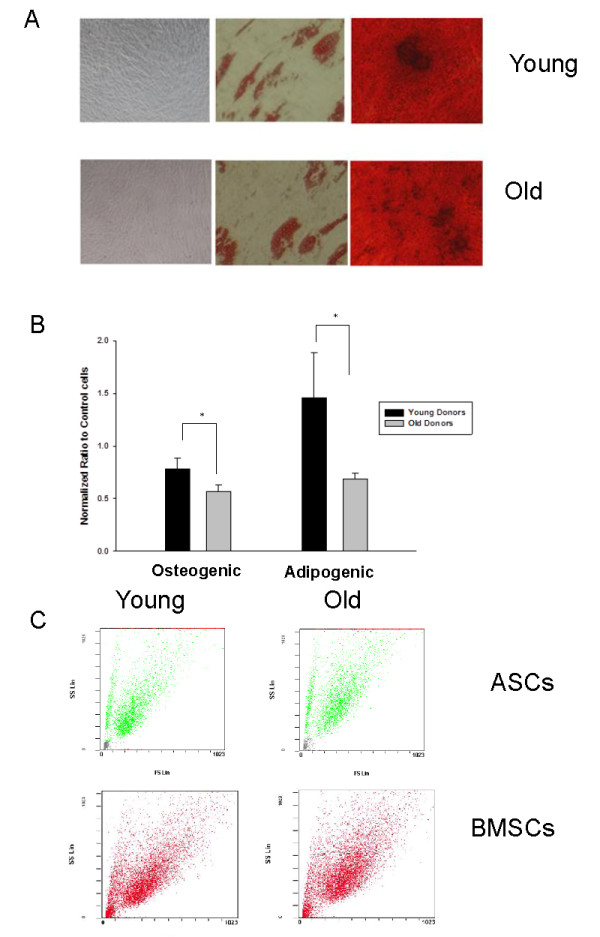
**Characterization of age-dependent differences in adipose stem cells (ASCs) and bone marrow stem cells **(**BMSCs)**. **(a) **Representative photomicrographs of MSCs show undifferentiated control cells (left), lipid inclusions with Oil Red O (middle), and mineralization with Alizarin Red (right) in ASCs from young (top) and old (bottom) donors. Original magnification of undifferentiated and mineralized cultures was ×10, and lipid inclusions were at ×40. **(b) **Quantification of differentiation potentials of ASCs from young and old donors shown as OD normalized to protein for cells cultured in osteoblastogenic and adipocytogenic conditions. Bars represent mean ± SEM and are representative of three quantifications. *P < 0.05. **(c) **A representative plot from flow cytometry shows forward versus side light scatter used to assess cell size and heterogeneity of MSCs. Green plots demonstrate ASCs, and red plots indicate BMSCs. Data for young donors are on the right, and for old donors, on the left.

ASCs and BMSCs were grown in specific culture media to determine their ability to differentiate along osteogenic and adipogenic lineages. MSCs, collectively both ASCs and BMSCs, from each age group demonstrated bone mineralization and neutral lipid accumulation in the appropriate culture medium and conditions, thus confirming the multipotent nature of the MSCs (Figure 1a). Quantification of differentiation based on histochemical staining showed significantly less mineralization (Alizarin Red) and lipid production (Oil Red O) in MSCs from older donors (50 years or older) than in MSCs from younger donors (50 tears or younger) (*P *< 0.05); these data indicate that the differentiation potentials of MSCs are associated with the biologic age of the donor (Figure 1b). Specifically, bone mineralization of cultures of MSCs from younger donors was 1.4-fold that for MSCs from older donors. Similarly, adipogenesis in MSCs from younger donors was 2.3-fold that for MSCs from older donors. Undifferentiated cells were characterized with flow cytometry for the presence of commonly identified cell-surface markers for MSCs and were consistent with the commonly accepted profile. No discernible differences were found in stromal cell-surface marker profiles between donors based on age and cell type based on tissue of origin (data not shown). Assessment of forward versus side light scatter revealed no significant age-related differences in cell size for ASCs and BMSCs (Figure 1c).

### Changes in the miRNA profiles of ASCs and BMSCs secondary to biologic aging

**Figure 2:**
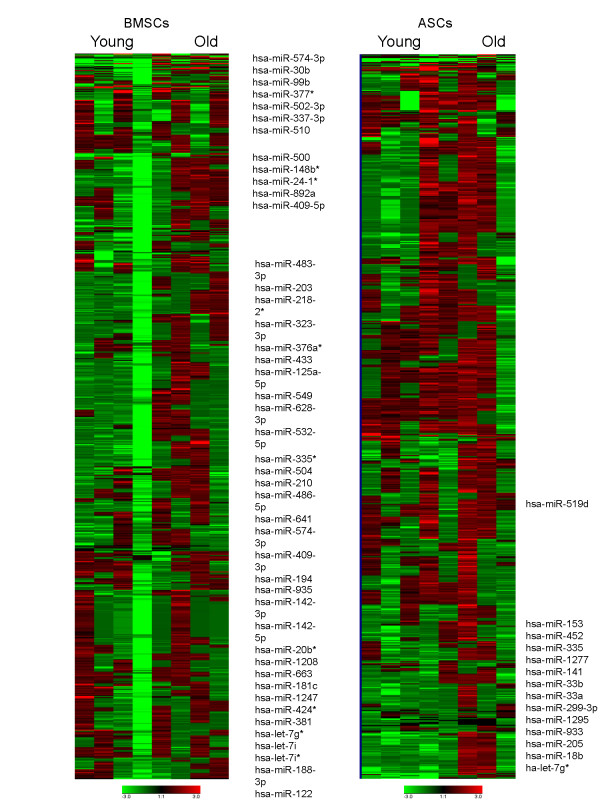
**Heatmap and hierarchic clustering of miRNA expression in adipose stem cells (ASCs) and bone marrow stem cells (BMSCs)**. **(a) **Expression of miRNA in old and young BMSC donors, and mapping of hierarchic clustering of miRNA and heatmap display of miRNA profiles. **(b) **Expression of miRNA in old and young ASC donors, and mapping of hierarchic clustering of miRNA and heatmap display of miRNA profiles. Columns represent individual donor samples, and each row represents individual assayed miRNA. Green represents downregulation of miRNAs, and red represents upregulation of miRNAs.

**Figure 3:**
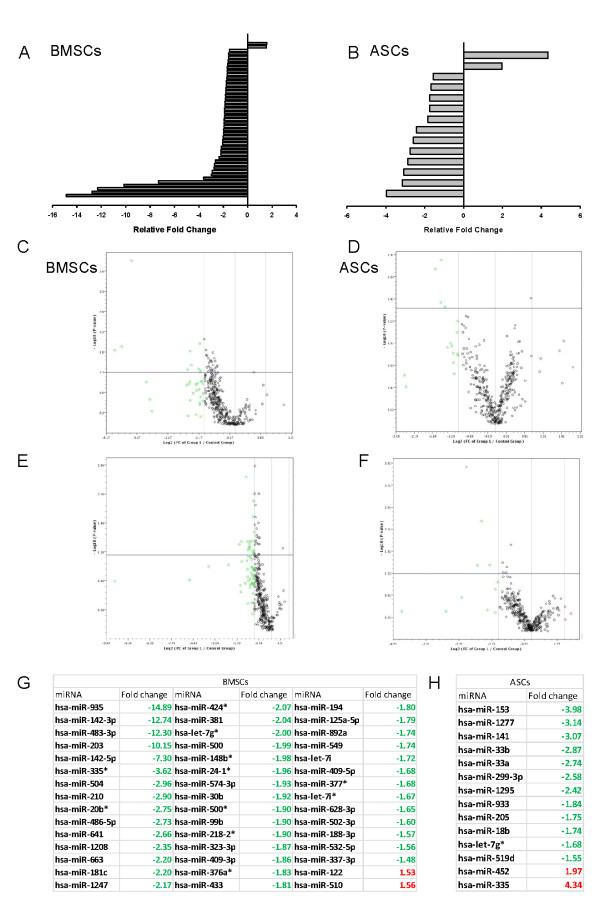
**Age-dependent changes in miRNA profiles of adipose stem cells (ASCs) and bone marrow stem cells (BMSCs). (a) **Statistically significant up- and downregulated miRNA in BMSCs. **(b) **Statistically significant up- and downregulated miRNA in ASCs. **(c, d) **Volcano plot of miRNA exhibiting *P *< 0.05 in expression as a result of BMSCs donor age. **(e, f) **Volcano plot of miRNA exhibiting *P *< 0.05 in expression as a result of ASCs donor age. **(g) **Fold regulation of significant miRNA in old versus young BMSCs donors. **(h) **Fold regulation of significant miRNA in old versus young ASCs donors. Upregulated miRNA are denoted in red, downregulated miRNAs are green, and miRNAs not statistically significant are black. Horizontal blue line represents *P*-value cutoff (*P *< 0.05), and vertical grey lines represent fold-change cutoff (more than twofold).

The miRNA profiles of ASCs and BMSCs from older and younger donors were analyzed with the qPCR-based array for miRNA of the whole human genome (Figure 2). Age-specific analysis showed significant differences (*P *< 0.05 compared with internal controls) in 45 miRNAs in BMSCs; these constituted approximately 5.86% of all evaluated human miRNAs (Figure 3a). For ASCs, significant age-related differences appeared in expression of 14 of 768 miRNAs, constituting 1.82% of all screened targets (Figure 3b). For both ASCs and BMSCs, greater numbers of miRNAs were downregulated in specimens from older donors than from younger donors (Figure 3c-f). The BMSCs showed that more than 95% of the significantly changed miRNAs were downregulated with age. ASCs had more than 85% of significantly changed miRNA downregulated with age. Specifically, 43 miRNA and 12 miRNAs in BMSCs and ASCs, respectively, were downregulated with age. Interestingly, ASCs and BMSCs each had two unique miRNAs among those screened that were significantly upregulated in older donors (Figure 3g, h).

### MicroRNA target prediction and bioinformatics assessments

**Figure 4:**
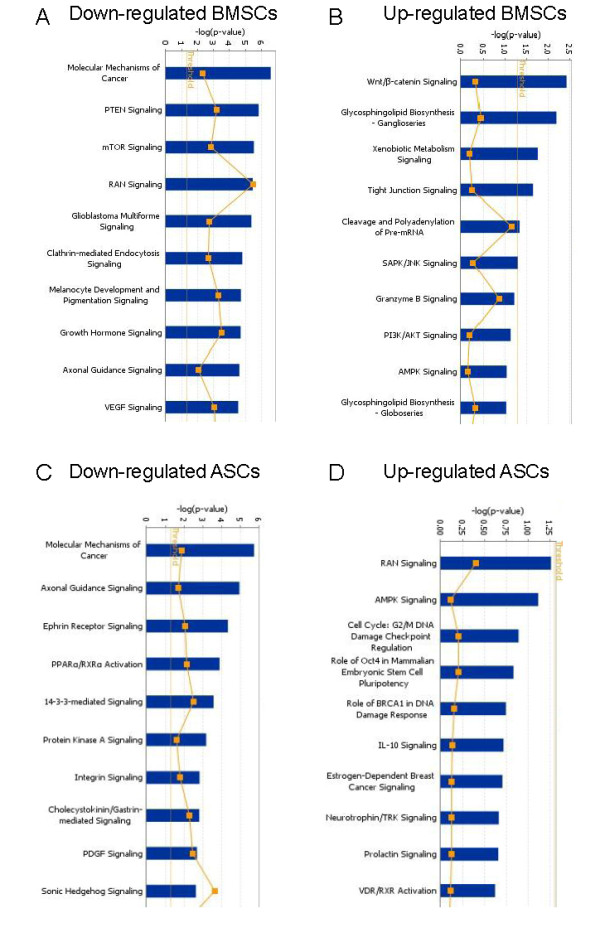
**Canonic pathway of predicted gene expression influenced by miRNA in adipose stem cells (ASCs) and bone marrow stem cells (BMSCs)**. Canonic pathways derived from predicted putative targets of miRNA action on gene expression by using Ingenuity Pathways Assessment (IPA) analysis of MSCs from old versus young donors. **(a) **Downregulated miRNA in BMSCs from old compared with young donors. **(b) **Upregulated miRNA in BMSCs from old, compared with young donors. **(c) **Downregulated miRNA in ASCs from old, compared with young donors. **(d) **Upregulated miRNA in ASCs from old, compared with young donors. Yellow line represents *P*-value cutoff (*P *< 0.05), and yellow dots represent ratio of projected involvement of targets to actual inputted involvement. Height of bars is determined by projected involvement of particular pathway.

Lists of predicted genes associated with significant miRNAs differentially expressed secondary to age-related differences within a subset grouping (for example, downregulated BMSCs in older versus younger donors) were generated from TargetScan (data not shown). IPA was then used to generate canonic pathway involvement, biologic functions, and network analysis for the predicted targets. Canonic pathways generated for up- and downregulated miRNAs in BMSCs from older as compared with younger donors demonstrated the involvement of many pathways (Figure 4a, b). The pathways associated with the largest age-related decreases in miRNA levels of BMSCs (*P *≤ 0.05) included those involved in molecular mechanisms of cancer, PTEN signaling, mTOR signaling, and RAN signaling. It would be predicted that those pathways would have increased activity because the inhibitory miRNA levels were downregulated. Among the canonic pathways associated with the largest age-related increases in miRNA of older donor BMSCs were Wnt/β-catenin signaling, tight-junction signaling, cleavage and polyadenylation of pre-mRNA, and SAPK/JNK signaling; these observations suggest decreased activity of those component molecules in the older BMSCs. Analysis of downregulated miRNAs in ASCs revealed significant involvement of the canonic pathways, including molecular mechanisms of cancer, axonal guidance signaling, ephrin receptor signaling, and PPARα/RXRα activation (Figure 4c, d). The canonic pathways associated with the largest age-related increases in miRNA levels of older donor ASCs included RAN signaling, AMPK signaling, and cell-cycle regulation; however, none achieved the *P*-value threshold.

**Figure 5:**
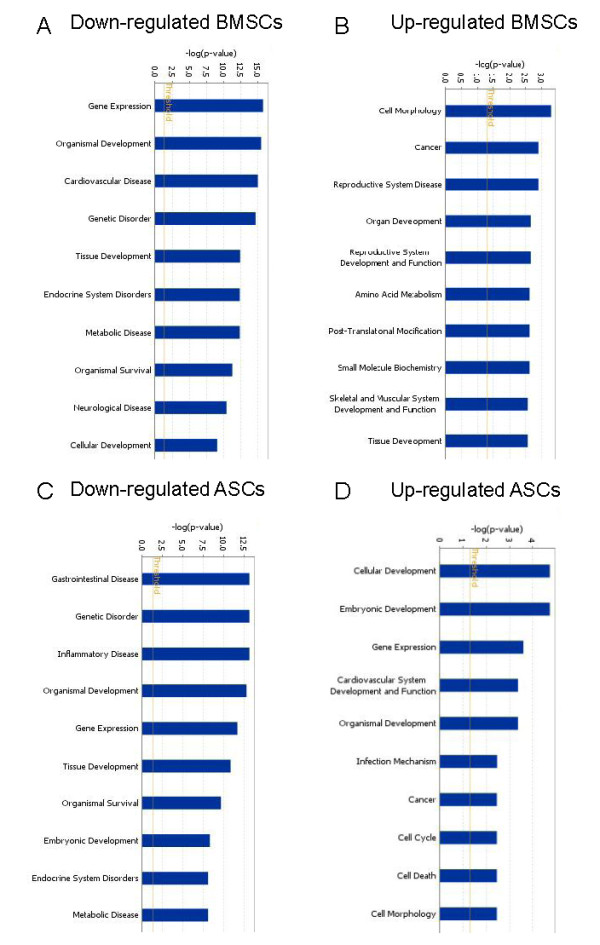
**Predicted biologic functions influenced by miRNA expression in adipose stem cells (ASCs) and bone marrow stem cells (BMSCs)**. Major biologic functions influenced by miRNA action on gene expression are shown from Ingenuity Pathways Assessment (IPA) analysis of MSCs from old and young donors. Involvement of biologic functions is determined from network and focus molecule association with miRNA targets in IPA. **(a) **Downregulated miRNA in BMSCs from old donors, compared with young donors. **(b) **Upregulated miRNA in M-MSCs from old donors, compared with young donors. **(c) **Downregulated miRNA in ASCs from old donors, compared with young donors. **(d) **Upregulated miRNA in ASCs from old donors, compared with young donors. The yellow line represents *P*-value cutoff (*P *< 0.05).

Subsequently, the gene lists of putative targets were analyzed with IPA software for biologic functions manifested secondary to age-dependent miRNA expression in MSCs. For age-related decreased miRNA targets of BMSCs, the top functions mapped included increased gene expression, organismal development, and cardiovascular disease (Figure 5a). For age-related increased miRNA targets in BMSCs, those with inhibition of biologic functions included cell morphology, cancer, and diseases of the reproductive system (Figure 5b). For ASCs, age-related decreased miRNA targets predict greater involvement of gastrointestinal disease, geriatric disorders, and inflammatory disease states (Figure 5c). For age-related increased miRNA targets in ASCs, the biologic functions that would be inhibited included cellular development, embryonic development, and gene expression (Figure 5d).

**Figure 6:**
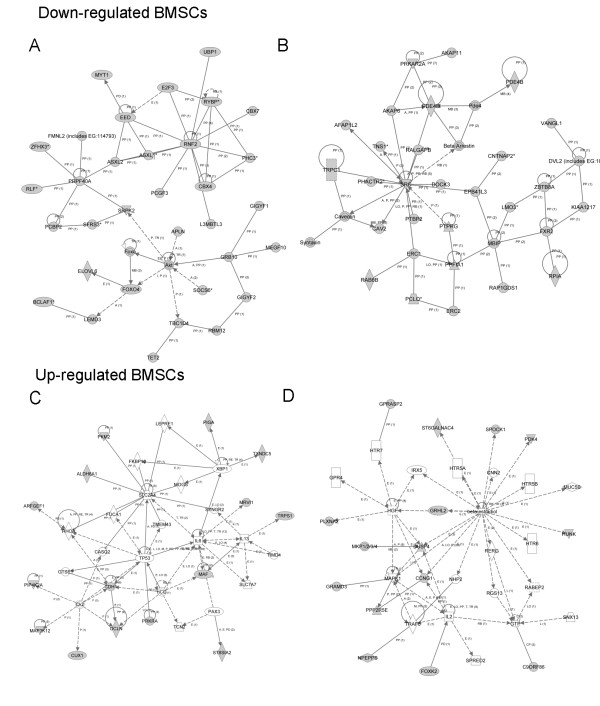
**Top network analysis of focus molecules by Ingenuity Pathways Assessment **(**IPA) in bone marrow stem cells **(**BMSCs)**. **(a, b) **Top two networks of focus molecules generated by IPA in direct or indirect regulation of gene expression by downregulated miRNA in BMSCs. **(c, d) **Top two networks of focus molecules generated by IPA of directly and indirectly involved gene-expression regulation by upregulated miRNA in BMSCs. Gray symbols, directly involved molecules, those inputted into IPA; white symbols, indirectly involved molecules, those commonly associated with the genes/pathways demonstrated.

**Figure 7:**
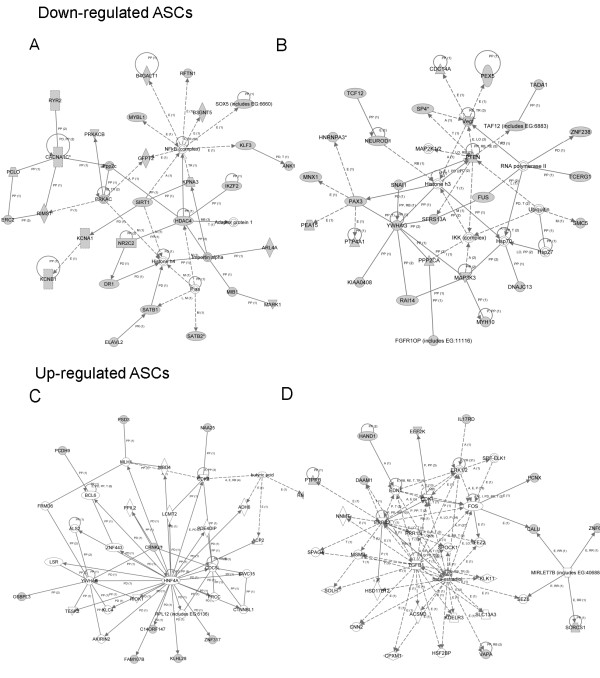
**Top network analysis of focus molecules by Ingenuity Pathways Assessment (IPA) in adipose stem cells (ASCs)**. **(a, b) **Top two networks of focus molecules generated by IPA in direct or indirect regulation of gene expression by downregulated miRNA in ASCs. **(c, d) **Top two networks of focus molecules generated by IPA of directly and indirectly involved gene-expression regulation by upregulated miRNA in ASCs. Gray symbols, directly involved molecules, those inputted into IPA; white symbols, indirectly involved molecules, those commonly associated with the genes/pathways demonstrated.

From the data for canonic pathways and biologic functions, IPA assessed networks of focus molecules, which were then grouped for the generation of networks of directly and indirectly involved molecules (data not shown). For age-related decreased miRNA in BMSCs, the major networks were related to cellular movement, cell signaling, cell death, and inflammatory diseases (Figure 6a, b). For age-related increased miRNA in BMSCs, major network functions involved cellular compromise, antigen presentation, cellular growth and proliferation, cell death, and cancer (Figure 6a, b). For age-related decreased miRNA in ASCs, the major significant networks were related to cell signaling, molecular transport, cell cycle, and gene expression (Figure 7a, b). For age-related increased miRNA in ASCs, major network functions were related to the cell cycle, cell-to-cell signaling and interaction, and cellular development (Figure 7a, b). The focus molecules were mapped by IPA, which generated networks of interaction between focus molecules and predicted or commonly associated target molecules. Major network pathways were created for up- and downregulated ASCs and BMSCs. Collectively, canonic pathways generated by IPA and analysis of the focus molecules networks reveal potential targets that miRNA differentially expressed in MSCs aging. Comprehensive evaluation of these major focus molecules from IPA revealed that miRNAs influencing NF-κB, ERK1/2, and IκB were directly involved.

### Age-related differences in constitutive mRNA expression in MSCs secondary to miRNA regulation

**Figure 8:**
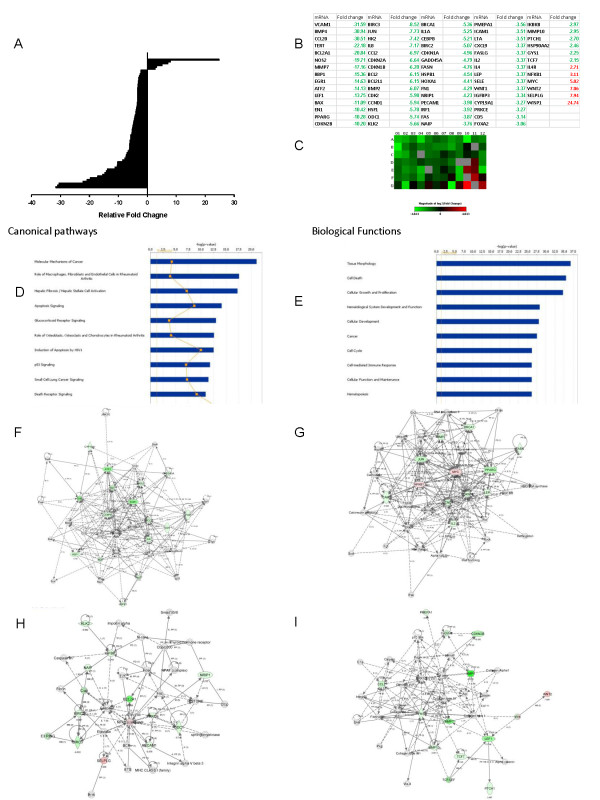
**Evaluation of miRNA influence on mRNA levels in adipose stem cells (ASCs) from old and young donors**. **(a) **The plot demonstrates significant up- and downregulated mRNA in ACSs from old versus young donors. **(b) **Fold regulation of significant mRNA assessed in ACSs from old versus young donors. **(c) **Heatmap shows old versus young donor mRNA expression levels; green represents downregulation of mRNAs, and red represents upregulation of mRNAs. (d) The plot shows the top canonic pathways generated from Ingenuity Pathways Assessment (IPA) analysis of mRNA differences in ACSs from old versus young donors. Yellow line, *P*-value cutoff (*P *< 0.05), and yellow dots, ratio of projected involvement of targets to actual inputted involvement. Height of bars is determined by projected involvement of particular pathway. **(e) **The plot shows top predicted biologic functions from the mRNA data inputted into IPA. Yellow line, *P*-value cutoff (*P *< 0.05). **(f **through **i) **The top four networks involved in mRNA differences between ACSs from old versus young donors. White items, indirectly involved molecules, those commonly associated with the genes/pathways demonstrated. Green items, downregulated molecules directly entered into IPA. Red items, upregulated molecules based on fold change of mRNA.

Representative ASCs from both age groups were selected based on microarray data clustering for further evaluation of the predicted effects of miRNA on constitutive mRNA expression. These representative ASCs were analyzed by qPCR-based signal-transduction array. This analysis confirmed that mRNA levels of the MAPK elements, iNOS, VCAM1, and IKK, as well as other NF-κB-pathway-related molecules were downregulated in ASCs from older donors compared with those from younger donors (Figure 8a through 8c). In contrast, mRNA for NF-κB and non-classically activated NF-κB targets, such as IL-4 receptor and *myc*, were significantly elevated. Other significantly upregulated mRNA in ASCs from older donors included WNT/β-catenin pathway constituents. Analysis of mRNA levels by using IPA showed similar canonic pathways and biologic functions as predicted from the miRNA data (Figure 7d through 7e). Analysis of focus molecules showed that major components of the MAPK/ERK pathway were downregulated in older donors and predicted depressed expression of related molecules. Furthermore, although the array showed elevated levels of NF-κB mRNA that IPA predicted based on the array data, other traditionally associated components of the inflammatory cascade were downregulated (Figure 8f through 8i). The exception to this was tumor necrosis factor-α (TNF-α), which IPA predicted would be increased due to a downregulation of miRNAs that act on it. Evaluation of mRNA levels of TNF-α showed increased levels; the fold difference compared with younger donors, however, was not significant. IPA and the Ingenuity Knowledgebase analysis and the mRNA array also showed inhibition of cell-cycle regulators such as cyclin-dependent kinases in ASCs from old donors, compared with young donors (Figure 8f through 8i).

### Age-dependent expression of MAPK/ERK and NF-κB in MSCs

**Figure 9:**
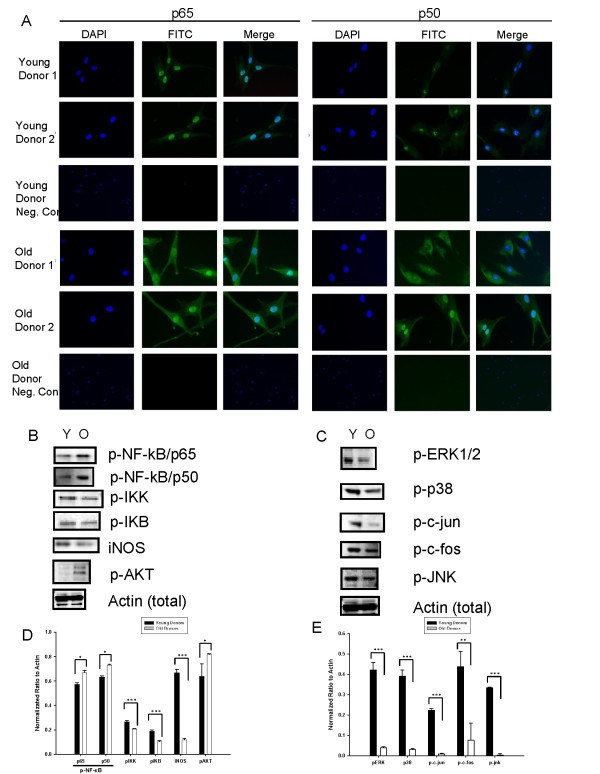
**Western blot analysis of differences in protein levels resulting from donor age and miRNA influences**. **(a) **Immunocytochemical localization of p65 and p50 subunits of NF-κB in representative ASCs from old and young donors. Images are at ×20 magnification. Negative controls are representative images per age group. Images are at ×5 magnification. **(b) **Representative Western blots of NF-κB pathway elements in ASCs from young (Y) and old (O) donors. **(c) **Representative Western blots of MAPK/ERK pathway elements in ASCs from young (Y) and old (O) donors. **(d) **The quantitative fold change in expression of NF-κB pathway proteins normalized to total actin levels. **(e) **The quantitative fold change in MAPK signaling expression of proteins normalized to total actin levels. Bars, mean ± SEM of at least three Western blot quantifications. *P < 0.05; **P < 0.01; ***P < 0.001).

Further evaluation of protein expression showed significant differences in detectable levels of screened molecules, as predicted from the miRNA data. Immunocytochemical localization of p65 and p50 subunits of NF-κB showed differential expression in cells from older and younger donors (Figure 9a). In cells from younger donors, NF-κB appeared to localize predominantly within the nucleus. In cells from older donors, however, detectable NF-κB was present in the nucleus, but dramatically elevated levels were observed in the cytoplasm. The pattern of distribution was similar for both p65 and p50 subunits of NF-κB, but was much more pronounced for p50. As assessed with Western blot, levels of phosphorylated inhibitory kappa B (p-IκB) (*P *< 0.001), phosphorylated inhibitory kappa B kinase (p-IκK) (*P *< 0.001), and inducible nitric oxide (iNOS) (*P *< 0.001) were significantly decreased in ASCs from older donors, compared with those from younger donors (Figure 9b, d). Interestingly, this analysis showed that phosphorylated NF-κB/p65 (p-NF-κB) levels were significantly elevated in ASCs from older donors as compared with younger donors (*P *< 0.05). Furthermore, as predicted by miRNA screening and mRNA data, Western blot analysis showed that levels of phosphorylated ERK1/2 (p-ERK1/2) (*P *< 0.01), phosphorylated c-fos (p-c-fos) (*P *< 0. 01), phosphorylated c-jun (p-c-jun) (*P *< 0.001), and phosphorylated JNK (p-JNK) (*P *< 0.001) were all significantly decreased in ASC from older donors compared with younger donors (Figure 9c, e).

## Discussion

In the present study, the comprehensive miRNA profiles of undifferentiated ASCs and BMSCs were analyzed to identify age-related differences in constitutive potential biologic functions at the mRNA and protein expression levels. The results showed that in MSCs isolated from two distinct tissues/organs, small subsets of miRNAs display age-related differences in the regulation of gene expression involving specific cellular and molecular pathways of cell proliferation and inflammation, presumably as regimented markers of aging. Specifically, significant age-related decreases were found in the MAPK/ERK pathway and NF-κB pathway expression.

Originally, it was thought that the hallmark of MSCs was their ability to differentiate into various end-cell types for regenerative repair of damage due to injury from disease processes [11,35,36]. Subsequently, many reports suggested that the role of MSCs as treatment modalities may occur through the expression of cytokines and chemokines that promote angiogenesis or squelch inflammatory responses, and adhesion molecules that would subsequently induce endogenous repair of injured cells and tissues [7]. In addition to these mechanisms that MSCs use to ameliorate cellular damage, their differentiation capabilities remain an important defining characteristic. We confirmed that ASCs from young donors, compared with those from older donors, had significantly elevated potential to differentiate toward osteogenic and adipogenic lineages. Other studies found similar changes while investigating aging of MSCs from humans, mice, rhesus macaques, and cynomolgus monkeys [3,30,37,38]. Our flow-cytometry results failed to detect changes in cell-surface markers secondary to the aging process. Thus, despite other age-related changes in MSCs, the cells can be identified by using cell-surface markers frequently used to distinguish MSCs from other cell types, regardless of the age of the donor [27].

Analysis revealed that individual targets selected from the IPA analysis followed the predicted pattern of expression at the mRNA and protein levels. All evaluated upregulated miRNA targets showed decreased levels of both mRNA and functional protein, including those involved in the MAPK/ERK and NF-κB pathways and in cell-cycle control. This is in accordance with recent results suggesting that although miRNAs promote both mRNA degradation and posttranslational repression, they appear to act predominantly as regulators at the mRNA level [19]. Furthermore, the current findings suggest that the miRNA profile of MSCs can be used to identify potential signaling mechanisms involved in MSCs functions, and perhaps even provide another way to classify MSCs function.

Several recent reports have demonstrated the function of the let-7 family of miRNA and its role in MSCs, especially as markers and regulators of a differentiated state [39]. The let-7 family was shown to repress cellular replication and inhibit self-renewal [40]. Our data showed that let-7 family miRNA were significantly downregulated in both ASCs and BMSCs from older donors. Accordingly, it can be surmised that ASCs and BMSCs in older donors may not be as efficient at self-renewal and would have decreased overall proliferation, compared with younger donors. Other reports determined that human skeletal muscle cells from older donors demonstrate reduced expression of let-7 and decreased mRNA levels of cell-cycle regulators such as CDK6 [25]. Our analysis of mRNA levels of cell-cycle regulatory molecules found that multiple cyclin-dependent kinases were downregulated, contributing to a reduction in cell proliferation. The data also showed increased miRNA targeted toward the Wnt/β-catenin-signaling pathway. Recent studies have shown that prolonged activation of Wnt signaling promotes MSC proliferation and contributes to aging [41]. Thus, our results demonstrate that miRNA inhibits Wnt/β-catenin signaling to decrease cell proliferation in aged MSCs, and potentially plays a role in retarding the aging process in MSCs.

The miRNAs directed toward the MAPK/ERK system were expressed at higher levels in cells from older donors. Specifically, ERK1/2 and JNK gene expression were involved as putative targets for miRNA-mediated gene-expression control. The downregulation of mRNA levels for c-fos and c-jun were confirmed by using real-time PCR and, by Western blot, demonstrated decreased protein levels of the MAPK pathway. Collectively p38, p-ERK1/2, p-c-fos, p-c-jun, and p-JNK levels were all significantly reduced in the ASCs of older donors as compared with those of younger donors, with the extension to BMSCs due to a similar miRNA profile and IPA analysis. Previous studies have indicated that BMSCs from older donors have decreased proliferation potential [2,42,43]. Additional reports have suggested that the dynamics of the aging process of MSCs is a determinant of cellular aging; however, the exact mechanism remains unclear [10]. The identified differences in miRNA in cells from older donors may represent the mechanism by which MSCs, through control over the MAPK/ERK signaling cascade, decrease cellular proliferation rates, thereby contributing to reduced tissue renewal in aging.

Although many of the fine details of aging in humans are yet to be elucidated, the interplay of aging and inflammation has been intensively researched. Many abnormalities in cellular processes have been found to occur with aging, including the development of cancer and type II diabetes mellitus [17,44]. At the center of these disease processes lie the common denominators of advanced age and inflammation. Interestingly, elevated levels of activated NF-κB were observed in older-donor MSCs. Whereas regulatory and traditional components of the NF-κB pathway, including, among others, IκB, IκK, iNOS, and IL-1α, were downregulated, other nontraditionally associated molecules were upregulated, including IL-4 receptor and *myc *oncogene. Traditionally, protein factors responsive to NF-κB transcriptional regulation would further amplify NF-κB expression, which was not observed in the current study [45]. In addition, the subcellular distribution of NF-κB observed in MSCs from older donors suggests the possibility of an alternative role in MSCs aging. Aging and NF-κB activation may have more in common than was initially postulated. Previous reports indicated that NF-κB can function in resistance to apoptosis [46]. In addition, NF-κB was shown to repress apoptosis triggered by JNK [47]. These observations, combined with our data showing decreased levels of JNK, provide evidence that NF-κB may be central to a protective role in MSC aging. Indeed, our data demonstrate that elevated levels of NF-κB regulated by miRNA activity may play a central role in the onset and progression of the aging process in MSCs.

The results also suggest that a delicate balance is maintained as a result of increased NF-κB expression in older MSCs. Typically, elevated levels of NF-κB are linked with pathologic processes; in old cells, however, elevated levels of NF-κB could prevent apoptosis [45]. Prevention of apoptosis would be of substantial importance to MSCs, especially given their function for endogenous cellular repair. Most studies, however, have demonstrated a potent anti-inflammatory role for MSCs [7]. The paradigm shift occurs with the notion of how a potently anti-inflammatory cell would maintain elevated levels of NF-κB. Our results demonstrated that although NF-κB levels are indeed elevated in older donors, presumably to prevent activation of apoptotic pathways, other commonly associated molecules in the NF-κB and inflammatory cascade were downregulated. IPA analysis, mRNA expression, and protein levels demonstrated that molecules such as IL-1α, TNF-α, iNOS, and IκK were significantly downregulated in ASCs from older donors. In addition, JNK was also significantly downregulated in older donors, giving support to the role of NF-κB as an inhibitor of apoptosis. That IκB levels decreased in the older donor ASCs is indicative of prolonged NF-κB activity. Although the classic NF-κB activity simultaneously causes *de novo *synthesis of IκB, it appears that NF-κB levels that are elevated because of aging in MSCs do not work through this mechanism. Intracellular localization of NF-κB from the current study in cells from younger donors appeared more predominant in the nucleus, specifically in the nucleolus, whereas cells from older donors demonstrated accumulation of NF-κB in the cytosol; these observations suggest that although it was phosphorylated in both groups, NF-κB was functioning differently as a function of age. Alternatively, relocalization of NF-κB subunits to the cytosol may indicate a lack of transcriptional activity, as further evidenced by decreased pro-inflammatory cytokines and other molecules including IL-1α, IL-8, and iNOS mRNA levels in cells from older donors compared with younger donors. Further investigation of the role of elevated NF-κB levels is required to gain a full understanding of the mechanisms at work in the MSC aging process.

## Conclusions

In summary, miRNA profiling demonstrated that subsets of miRNAs are biologically active in human MSCs, with the profiles of miRNAs changing with aging in both ASCs and BMSCs. Furthermore, miRNAs modulate and regulate gene expression related to a variety of functions, particularly cellular proliferation and inflammation, both of which play an integral role in the process of aging. ASCs from older donors also exhibit significantly elevated levels of NF-κB, perhaps in response to downregulation of the MAPK/ERK system to prevent proliferation and inhibit apoptotic stimuli. Interestingly, other molecules commonly associated with NF-κB were downregulated, leading to a unique and novel constellation of expression of the core inflammatory molecules in a potently anti-inflammatory cell. The current study presents, to our knowledge, the first report that miRNAs have unique roles in the aging of MSCs, maintaining their capacity to suppress inflammation and promote endogenous cellular repair while, perhaps, allowing MSCs to escape the aging process.

## Abbreviations

ASCs: adipose-derived mesenchymal stem cells; BMSCs: bone marrow-derived mesenchymal stem cells; CCM: complete culture medium; IPA: Ingenuity Pathway Assessment; MAPK: mitogen activated protein kinase; mRNA: messenger ribonucleic acid; miRNA: micro ribonucleic acid; MSCs: mesenchymal stem cells; NF-κB: nuclear factor kappa B; PCR: polymerase chain reaction.

## Competing interests

JMG is the Founder of LaCell, LLC, and serves as a consultant for Johnson & Johnson companies ATRM and Mentor; he has also received fees from Toucan Capital and its subsidiary companies in the adipose stem cell area. The other authors declare that they have no competing interests.

## Authors' contributions

ACP and BAB conceived, designed, and executed the experiments. ACP, JS, and RPO characterized the cells. ACP, DK, and BAB preformed bioinformatics and statistical analyses. ACP, BAB, and JMG analyzed the data. ACP and BAB wrote the article. JMG, JS, and JG edited the article. All authors read and approved the final manuscript.
